# The Impact of Mutations in the *HvCPD* and *HvBRI1* Genes on the Physicochemical Properties of the Membranes from Barley Acclimated to Low/High Temperatures

**DOI:** 10.3390/cells9051125

**Published:** 2020-05-01

**Authors:** Elżbieta Rudolphi-Szydło, Iwona Sadura, Maria Filek, Damian Gruszka, Anna Janeczko

**Affiliations:** 1 Institute of Biology, Pedagogical University, Podchorażych 2, 30-084 Kraków, Poland; elzbieta.rudolphi-szydlo@up.krakow.pl (E.R.-S.); mariafilek@gmail.com (M.F.); 2 Polish Academy of Sciences, The Franciszek Górski Institute of Plant Physiology, Niezapominajek 21, 30-239 Kraków, Poland; i.sadura@ifr-pan.edu.pl; 3 Institute of Biology, Biotechnology and Environmental Protection, Faculty of Natural Sciences, University of Silesia, Jagiellonska 28, 40-032 Katowice, Poland; damian.gruszka@us.edu.pl

**Keywords:** brassinosteroids, cell membranes, Langmuir monolayers, galactolipids, plant acclimation to high/low temperature, phospholipids

## Abstract

(1) Background: The study characterized barley mutants with brassinosteroid (BR) biosynthesis and signaling disturbances in terms of the physicochemical/structural properties of membranes to enrich the knowledge about the role of brassinosteroids for lipid metabolism and membrane functioning. (2) Methods: The Langmuir method was used to investigate the properties of the physicochemical membranes. Langmuir monolayers were formed from the lipid fractions isolated from the plants growing at 20 °C and then acclimated at 5 °C or 27 °C. The fatty acid composition of the lipids was estimated using gas chromatography. (3) Results: The BR-biosynthesis and BR-signaling mutants of barley were characterized by a temperature-dependent altered molar percentage of fatty acids (from 14:0 to 20:1) in their galactolipid and phospholipid fractions in comparison to wild-type (WT). For example, the mutants had a lower molar percentage of 18:3 in the phospholipid (PL) fraction. The same regularity was observed at 5 °C. It resulted in altered physicochemical parameters of the membranes (A_lim_, π_coll_, Cs^−1^). (4) Conclusions: BR may be involved in regulating fatty acid biosynthesis or their transport/incorporation into the cell membranes. Mutants had altered physicochemical parameters of their membranes, compared to the WT, which suggests that BR may have a multidirectional impact on the membrane-dependent physiological processes.

## 1. Introduction

During their life cycle, plants are exposed to changing environmental conditions—biotic (pathogenic microorganisms) or abiotic (extreme temperature, drought, flooding, nutritional depletion, too low or too intense light, UV radiation, etc.). Among the abiotic stresses, temperature stress is a particularly serious problem for crop production. Some species are sensitive to cold (cucumber, maize). Frost, on the other hand, especially with an insufficient snow cover on the fields, can cause significant yield losses of winter crops including winter cereals. High-temperature stress is particularly dangerous for plants when combined with a water deficit. Gradually increasing temperature allows plants to acclimate and tolerate further increases in temperatures, which normally might be lethal. On the other hand, cold can acclimate plants to freezing temperatures and in the winter plants, this is known as the cold-hardening (cold acclimation) process. During acclimation, metabolic adjustments occur in plant cells that include, among others, changes in the hormonal homeostasis, an elevated biosynthesis of the proteins with chaperone properties (i.e., heat shock proteins) or enhancement of the antioxidant system. Crucial changes that stimulate the acquisition of frost tolerance influence the cell membranes, especially in cold acclimation. Since membranes are considered to be “thermal sensors” (according to the membrane sensor hypothesis [1], they are thought to elicit other metabolic changes within a cell, including gene expression. In the lipid part of the cell membrane, acclimating to elevated temperatures or cold causes essential physicochemical adjustments, one of which is the modification of the fatty acid composition. Higher temperatures usually initiate a decrease in the level of unsaturated acids in the lipid part of a membrane [2]. In cold, the composition of the fatty acids of the membrane lipids is expected to become more unsaturated [3]. This unsaturation of the fatty acids causes a decrease in the phase transition temperature and increases the “fluidity” of the hydrophobic phase. The main fatty acids from groups 16C and 18C (especially 18:3 or 18:2), present in cells in the highest amounts, have a significant physiological role in plant response to abiotic but also biotic stresses [2,3]. In winter plants, low temperature stimulates biosynthesis of 18:3 acid, resulting in an increase of unsaturation of membrane lipids thus better acclimation to unfavorable conditions of growth in winter [3,4]. On the other hand, a lowered accumulation of 18:3 (and increased lipid saturation) is beneficial for instance for better thermostability of the photosynthetic apparatus at higher temperatures [2]. Polyunsaturated fatty acids can also be released from membranes in response to an attack by biotic agents [5,6,7]. Fatty acid 18:3 may directly activate NADPH-oxidase and generate reactive oxygen intermediates after inoculation with bacteria. On the other hand, lipid-derived metabolites produced by oxidation of fatty acids (18:3 or 18:2)—oxylipins like jasmonic acid—are an integral part of plant defense against pathogens.

Plant growth hormones/regulators such as brassinosteroids (BR) can improve the tolerance of plants to low- or high-temperature stress [8]. BR are plant steroid hormones that have been extensively studied during the past three decades. These studies have revealed many of the mechanisms of their action in the process of growth and development stimulation as well as plant stress tolerance [9]. However, little is known about the impact of BR on the plant membrane structure (lipid part) and the membrane physicochemical properties, which are, as was mentioned, important in the process of plants acclimating to various temperatures.

The effects of the exogenous administration of BR on the lipid FA composition in plants have been found in several previous studies [10,11,12,13]. Further, Li et al. [12] in studies using electron paramagnetic resonance observed an increase in the membrane fluidity in the presence of brassinolide in mango, which was an important step for developing an improved plant tolerance to low temperature. Our studies [14,15] showed that the structural properties of the cell membranes were differentiated by the presence of brassinosteroids, and therefore, the role of BR in improving the tolerance of winter wheat to low temperatures was suggested. In Langmuir monolayer studies, 24-epibrassinolide and 24-epicastasterone were introduced into lipids that had been obtained from the aerial part of winter wheat seedlings that had been cultured at 5 °C or 20 °C. It was suggested that the tested BR (similar to sterols) entered the cell membrane directly and modified its properties by, for example, increasing the distance between the fatty acid chains, which might improve the functioning (flexibility) of the membrane in low temperatures [14,15]. As 24-epicastasterone induced a slightly different effect than 24-epibrassinolide, these results also showed the importance of the chemical structure of BR for their interactions with membranes [14].

The novelty of the presented studies, contrary to studies with the exogenous BR treatments, was the use of barley BR-deficient and BR-insensitive mutants to verify how disturbances in the BR biosynthesis (mutation in the *HvCPD* gene) and the BR signaling (mutation in the *HvBRI1* gene) in barley change the FA lipid composition and the physicochemical properties of the cell membranes. We characterized the natural lipid monolayers that had been obtained directly from these barley mutants (with a decreased or increased content of endogenous BR), which had been acclimated at 5 °C and 27 °C. It is worth mentioning that our earlier studies [16] revealed that the mutants (after acclimation at 27 °C) had acquired a heat tolerance that was higher than the wild-type. After acclimation at 5 °C, both mutants had a lower frost tolerance. In our earlier articles, we also described changes in the protein component of the cell membranes (aquaporins, heat shock proteins) that might modify the mutants’ tolerance to high temperature or frost [17,18]. In the current work, we focused on the lipid part of the cell membranes. The aim was to investigate the dependence between the FA composition of the individual lipid fractions and changes in the physicochemical properties of the membrane structure, which determines their permeability, stiffness/fluidity, and ability to be penetrated by various compounds including hormones. The characteristics of the physicochemical properties of membranes are increasingly used to explain the subtle changes in the structure of lipids that occur during the physiological processes (review Rudolphi-Skórska and Sieprawska [19]). In the current work, this approach enabled (I) the dependence between an increased/decreased level of the endogenous BR in leaf tissue and structural-functional properties of membranes to be discussed and (II) the role of BR in the low/high-temperature tolerance mechanism that involves the modifications of the membranes to be deliberated.

## 2. Plant Material and Experimental Design

The BR-deficient mutant BW084 and the BR-insensitive mutant BW312, which are near the isogenic lines (NILs) of the wild-type (WT) Bowman cultivar, were selected for the studies. The homogenous genetic background simplifies comparative physiological analysis [20,21]. BW084 has a mutation in the *HvCPD* gene coding enzyme that mediates the early BR biosynthetic steps and the brassinolide or castasterone content in this mutant are significantly reduced when compared with the WT [16,22]. The BR-signaling mutant BW312 has a mutation in the gene that encodes the BR receptor *HvBRI1* and due to the BR-insensitivity accumulates more BR than the WT cultivar [16,22]. The barley seeds were obtained from the collection of the University of Silesia (Katowice, Poland). The plant cultures, growth conditions, and experimental design are described in detail in the work by Sadura et al. [16]. Briefly, after seed germination, the plants were transferred to pots (15–20 plants/pot) and then cultured in a growth chamber (16 h photoperiod, 20 °C) for about three weeks. When the plants had developed three to four leaves, the pots were divided into two groups. One group was acclimated at 5 °C (21 days, 8 h photoperiod), while the second was acclimated at 27 °C (16 h photoperiod) for seven days. The leaf samples for the analyses were collected from the plants before acclimation (20 °C) and after acclimation at 5 °C or 27 °C.

### 2.1. Lipid Extraction and Fatty Acid Composition Measurement

The lipids of the membranes were extracted from the leaves using a mixture of methanol/ chloroform (2:1) and were then re-extracted with chloroform according to a modified method of Bligh and Dyer [23], which was described in detail by Gzyl-Malcher et al. [24] and Janeczko et al. [10]. The lipid fractions, which included phospholipids (PL) and the glycolipids (monogalactosyldiacylglycerols [MGDG] and digalactosyldiacylglycerols [DGDG]), were separated on silica acid using column chromatography as was described in the work Janeczko et al. [10]. The purity of obtained fractions was checked by thin-layer chromatography (TLC). The fatty acid (FA) esterification was made based on a modified AOAC Official Method 991,39 [25]. The equipment for the analysis on the gas chromatograph were a chromatograph TRACE GC ULTRA (Thermo Electron Corporation, Milano, Italy), a flame ionization detector (FID), and a STABILWAX column (30 m—0.25 mm—0.25 μm). The individual fatty acid methyl esters were identified by comparing them to the standard mixture of Supelco 37 component FAME Mix (Sigma-Aldrich, Poznań, Poland). Fatty acids from 14:0 to 21:1 were detected (14:0—myristic acid; 16:0—palmitic acid; 16:1n-9—*cis*-7-hexadecenoic acid; 16:1n-7—cis-9-hexadecenoic (palmitoleic acid); 18:0—stearic acid; 18:1 n-9—oleic acid; 18:1 n-7—vaccenic acid; 18:2 n-6 (18:2)—linoleic acid; 18:3 n-3 (18:3)—linolenic acid; 20:0—arachidic acid and 20:1—eicosenoic acid). The data are expressed as the molar percentage of a specific fatty acid in relation to all of the fatty acids that were measured. The degree of the unsaturation of the individual lipid fractions was determined based on the calculated ratios of the unsaturated to saturated FA (U/S) and 18:3/18:2.

### 2.2. The Langmuir Monolayers and Their Physicochemical Parameters

Fractions of extracted polar lipids, i.e., PL, DGDG, and MGDG, were used for the experiments using the Langmuir technique (Minitrough, KSV; Finland). The monolayers were produced by spreading chloroform solutions of the lipids on the surface of the water (as the subphase). The Langmuir monolayers were compressed at a rate of 3.5–4.6 Å^2^/molecule × min. A Platinum Wilhelmy plate connected to an electrobalance was used to detect the surface pressure (accuracy of ±0.1 mN/m). All of the experiments were performed at 25 °C. The measurements were repeated three/four times to confirm a high recurrence of the obtained isotherms (±0.1–0.3 Å^2^). 

Based on the dependence of the surface pressure (π) versus the area per lipid molecule (*A*), the parameters that characterized the structure of the monolayers were A_lim_—the area that was occupied by a single molecule in a completely packed layer and π_coll_—the value of the surface pressure at which a layer collapsed. Moreover, the static compression modulus was calculated as C_s_^−1^ = −(d**π** /dlnA). This parameter indicates the mechanical resistance of the layers and provides information on the stability and fluidity of a layer.

### 2.3. Statistical Analysis

**Figure 1:**
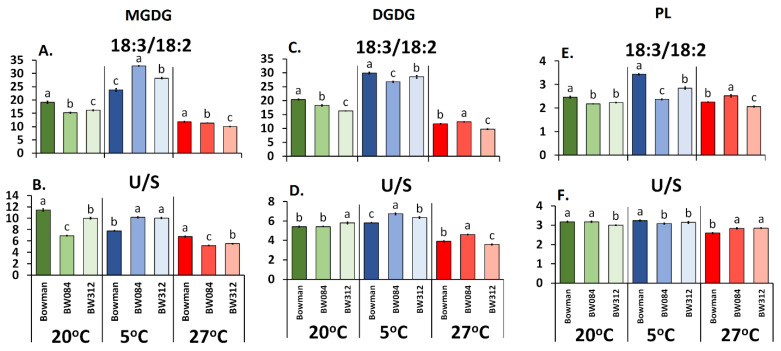
The ratio of fatty acid 18:3 to 18:2 (18:3/18:2) and unsaturated fatty acids to saturated fatty acids (U/S) for the specific lipid fractions (galactolipids and phospholipids) that were isolated from the leaves of the barley WT Bowman, mutant BW084 and mutant BW312 cultured at 20 °C and then acclimated at 5 °C and 27 °C. Any significant differences between WT Bowman and its mutants (Duncan test, *p* ≤ 0.05) for each temperature are indicated by different letters. (**A**,**B**) Fraction of galactolipids (MGDG); (**C**,**D**) Fraction of galactolipids (DGDG); (**E**,**F**) Fraction of phospholipids (PL).

Statistical analysis (ANOVA, post hoc test) was performed in Statistica 13.1 (StatSoft, Tulsa, USA). Duncan’s test was used to compare the averages of data obtained for the WT Bowman, BW084, and BW312 (Figures 1 and 3, Table 1). Values marked with the same letters did not differ significantly (*p* ≤ 0.05). Values on Figures 1, 3 and Figure S1—supplementary materials are given ±SD.

## 3. Results

### 3.1. Fatty Acid Composition of the Membrane Lipids from Barley Growing at 20 °C and the Physicochemical Properties of the Lipid Monolayers

Focusing on fatty acid 18:3 (PL fraction), which was present in the highest percentage (approx. 50% of the FA pool), it can be observed that the amount of this acid was significantly lower at 20 °C in both tested mutants, BW312 and BW084, compared to the WT Bowman (Table 1). This phenomenon was accompanied by an increased content of 18:2. The content of the third acid (saturated 16:0) in the mutants at 20 °C was higher (BW312) or remained unchanged (BW084). The level of 18:0, present, however, at a low percentage, was increased in the signaling mutant, but not in the BR-biosynthesis mutant. In the MGDG and DGDG fractions, the content of 18:3 acid was much higher than in the PL fraction and reached approximately 80%. The mutants in most cases also exhibited a lower content of 18:3 in these fractions compared to the WT Bowman. In all of the tested fractions, the 18:3/18:2 ratio was lower in the mutants compared to the WT (Figure 1A,C,E). The U/S ratio was lower in both of the mutants for the MGDG fraction (Figure 1B). The results were less evident for the DGDG and PL (Figure 1D,F).

Other FA were also detected in the PL, MGDG, and DGDG fractions: 14:0, 16:1, 18:1, 20:0, and 20:1 (Table 1). These FA were, however, present at a very low percentage (below 3% and much lower). At 20 °C (as also after acclimation at 5 °C and 27 °C), slight changes were observed within the molar percentage of these FA in the mutants compared to the WT.

**Figure 2:**
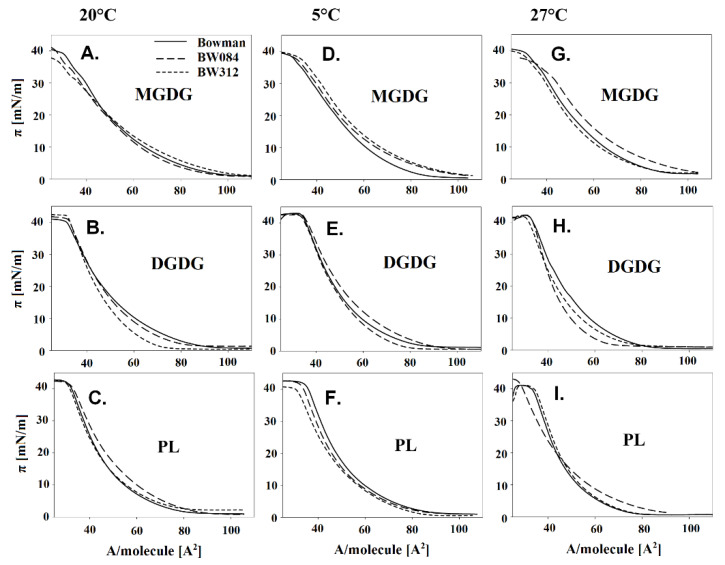
Exemplary Langmuir isotherms (surface pressure ***π*** vs area per molecule ***A***) for the monolayers of the galactolipids - MGDG (A, D, G), DGDG (B, E, H) and phospholipids - PL (C, F, I) that were obtained from the leaves of the barley WT Bowman, mutant BW084, and mutant BW312 cultured at 20 °C (**A**–**C**) and then acclimated at 5 °C (**D**–**F**) and 27 °C (**G**–**I**).

**Figure 3:**
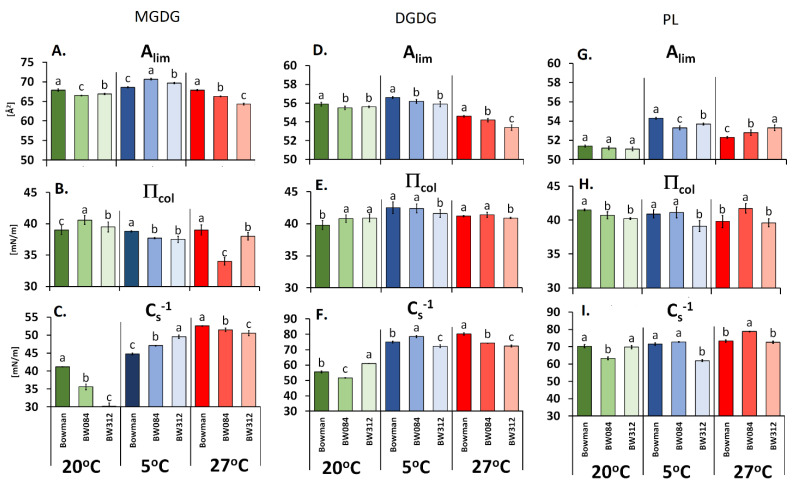
Physicochemical parameters: the limiting area per molecule (A_lim_ [Å^2^]), collapse pressure (π_coll_ [mN/m]) and compression modulus (C_s_^−1^ [mN/m]) of the monolayers that were prepared from the galactolipids - MGDG and DGDG (**A**–**F**) and phospholipids - PL (**G**–**I**) obtained from the leaves of the barley WT Bowman, mutant BW084, and mutant BW312 cultured at 20 °C and then acclimated at 5 °C and 27 °C. Any significant differences between WT Bowman and its mutants (Duncan test, *p* ≤ 0.05) for each temperature are indicated by different letters.

An analysis of the physicochemical properties of the membranes based on the dependence of the surface pressure (π) versus the lipid area (A) in individual fractions (Figure 2A–I) and the physicochemical parameters that were calculated based on these data (Figure 3A–I), it was found that for the plants growing at 20 °C, the values of A_lim_ in the PL fraction were generally similar both for the WT Bowman and its mutants, whereas for the galactolipids, the highest A_lim_ were obtained for the WT Bowman. The π_coll_ parameter significantly differentiated the lipids of the studied fractions that had been derived from the genotypes growing at 20 °C. The WT exhibited higher values of this parameter in the PL fraction and the lowest in galactolipids compared to mutants. The last parameter (C_s_^−1^) had the highest values for the BW312 in the PL fraction (although similar to the WT) and DGDG, while generally, the lowest values were for the MGDG lipids. 

### 3.2. Fatty Acid Composition of the Membrane Lipids from the Barley That Had Been Acclimated at 5 °C and the Physicochemical Properties of the Lipid Monolayers

Similar to 20 °C, the decrease in the 18:3 content in the FA pool of PL at 5 °C was accompanied by an increase in the 18:2 content in both mutants when compared with the WT Bowman (Table 1). At 5 °C, the content of 16:0 acid was higher in both mutants than in the WT. In the MGDG and DGDG fractions, the highest percentage was 18:3 (more than 80% of the FA pool). 

At the low temperature, the 18:3/18:2 ratio was higher in the mutants for the MGDG but lower for the DGDG and PL fractions compared to the WT (Figure 1A,C,E). The opposite effect was observed for the U/S (for DGDG). Value A_lim_ at 5 °C was higher in the mutants (MGDG fraction) and was accompanied by a higher C_s_^−1^ and lower π_coll_ values (Figure 3A–C). In the DGDG and PL fractions, the pattern of the changes of those three parameters was, with some exceptions, a little similar to that recorded at 20 °C. Especially for BW312, all of the values of A_lim_, π_coll,_ and C_s_^−1^ were at 5 °C always lower than in the WT. Figure S1A–I additionally illustrates the direction of the changes of the A_lim_, π_coll_, and C_s_^−1^ parameters in the mutants and the WT Bowman at 5 °C in relation to 20 °C (expressed as 100%). 

### 3.3. Fatty Acid Composition of the Membrane Lipids from the Barley Acclimated at 27 °C and the Physicochemical Properties of the Lipid Monolayers

Generally, the percentage of 18:3 in the PL fraction that was isolated from the plants acclimated at 27 °C was generally the lowest in all of the plants that were tested compared to 20 °C and the low temperature (Table 1). However, the level of this fatty acid was higher in the mutants compared to the WT, which was the opposite effect to that observed at 20 °C and 5 °C. The molar percentage of saturated 16:0 acid decreased in all of the mutants at 27 °C while the molar percentage of 18:2 decreased in the BW084 but increased in the BW312 (if compare to WT Bowman). In the MGDG and DGDG fractions, the content of 18:3 at 27 °C decreased in the BW312. As for BW084, the molar percentage of 18:3 was increased in DGDG but decreased in MGDG, in comparison to WT Bowman. The 18:3/18:2 ratios and U/S were generally the lowest for all of the galactolipid fractions compared to the values that were noted at 20 °C and 5 °C (Figure 1A–D). The 18:3/18:2 ratios and U/S were lower in the MGDG fraction in the mutants than in the WT. In DGDG, these ratios were higher in BW084 but lower in BW312 than in the WT. As can be seen in Figure 3A–F, the values of A_lim_, π_coll_, and C_s_^−1^ for the fraction of galactolipids were lower in the mutants than in the WT. The exception was π_coll_ in the BW084, which was similar to the WT in DGDG fraction. For the PL fraction, the A_lim_ value was higher in both mutants and it was accompanied by higher π_coll_ and C_s_^−1^ values compared to WT Bowman but only in the BW084. The changes of these physicochemical parameters (A_lim_, π_coll_, and C_s_^−1^) for the tested mutants and the WT Bowman, which were calculated additionally in relation to the temperature of 20 °C (100%), are presented in Figure S1A–I.

## 4. Discussion

Our work, which was devoted to mutants with the impaired biosynthesis and signaling of BR, permitted the results that have been obtained for fatty acids (especially 18:3) in the experiments in which BRs were applied exogenously to be partly confirmed [10,12,13]. Both the BR-deficiency and BR-signaling disorders in the mutants were reflected in the modification of the fatty acid composition/proportion in the individual lipid fractions. Earlier, Janeczko et al. [10] reported that the exogenous application of BR into a culture of oilseed rape calli increased the molar percentage of 18:3 in their PL fraction at 20 °C. In the current work, the mutants with the defects of BR biosynthesis and signaling growing at 20 °C had, as expected, a lower molar percentage of 18:3 in PL fraction. The same regularity was observed at 5 °C. The mutant BW084 had a lower molar percentage of 18:3 (in PL fraction), while BR-sprayed mango fruits, which subsequently were grown in the cold for 14–21 days, were characterized by an increased molar percentage of 18:3 in the polar lipid fraction [12]. Thus, the effects of an increasing BR level in tissues through its exogenous administration were, as expected, exactly the opposite of the effects that were caused by the BR deficit in the mutant. However, the proper functioning of the BR receptors was also important because, despite the increased BR level that is characteristic for the mutant BW312 [16,22], abnormal BR signaling often had a similar or the same effect as a BR deficiency. Although it appears that BR regulates the biosynthesis of the FA or their transport/incorporation into the cell membranes, this issue will require a more detailed investigation and explanation of the mechanism. The relationship between BR and FA was, of course, modified by the plant growth/acclimation temperature. Highly unambiguous results were obtained for the PL fraction (more characteristic for the plasma membrane) and 18:3 present within about 50% in the FA pool. Consistently, in both tested mutants, BR-biosynthesis or BR-signaling disorders were associated with a decrease in the content of this fatty acid in the PL fraction at 5 °C and 20 °C, but an increase in its content at 27 °C. It is assumed that for the better “adaptation” of membranes to temperature, the percentage of unsaturated FA should increase at 5 °C and decrease at 27 °C [26]. This is considered to be one of the steps in plant acclimation. Therefore, in the case of the PL fraction, the analyzed mutants had little less favorable parameters than the WT. The opposite situation we had observed in the galactolipid fractions. At 5 °C, the mutants had a higher percentage of 18:3 than the WT, while at 27 °C, the mutants were characterized by a lower content of 18:3, especially in the case of MGDG. Since galactolipids are more typical for the chloroplast membranes and it is known that the MGDG fraction constitutes about 55% of the thylakoid membrane lipids [27], this may partly explain why the mutants maintained better efficiency of photosystem II (PSII) at 5 °C and 27 °C when compared to the WT [16]. Moreover, studies on *Arabidopsis thaliana* mutants that carry mutations in the genes encoding the MGD-synthase also confirm the contribution of MGDG fraction to photosynthesis efficiency. These mutations led to a significant reduction in the ability of the mutant plants to conduct photosynthesis [28]. In our experiment more 18:3 (more fluidic thylakoid membranes) at low temperature and less 18:3 (less fluidic thylakoid membranes) at a higher temperature may provide a better environment to membrane-located processes at these temperatures, and PSII is located in the thylakoid membranes. In fact, as mentioned in the introduction, the accumulation of 18:3 is beneficial for the thermostability of the photosynthetic apparatus at higher temperatures [2]. Our earlier work [16] showed that mutants at 5 °C and 27 °C were characterized by higher (than WT) values of P.I._ABS_ informing about general PSII efficiency. In detail, mutants maintained comparable to WT plants energy transfer to electron transport chain (ETo/CSm) but it was accompanied by lower requirements of absorbed energy (ABS/CSm) and connected to lower energy loss as a heat (DIo/CSm).

According to a detailed analysis of the physicochemical properties of the lipid fractions, it seems that a greater amount of modifications between the tested plants were associated with the galactolipids rather than the PL fraction, and the MGDG fraction in particular. These results confirm the importance of the chloroplast structure in the plant response to the temperature changes. Thus, the physicochemical galactolipid modifications could be an important step, through FA composition changes, in the thylakoid membrane “adaptations” (stiffness/fluidity) that enable the proper functioning of photosystems at 5 °C and 27 °C. The value of the limiting area per molecule (A_lim_) usually increases at 5 °C compared to 20 °C (which was also visible in the lipid fractions of genotypes analyzed in our study (Figure 3A,D,G) and provides information about any increase in membrane fluidity [29]. It is worth noting that, in the main lipid fraction of thylakoids (MGDG), the increase in A_lim_—indicating the increase in membrane fluidity at 5 °C—was greater in the mutants than in the WT Bowman (Figure 3A). The opposite effects, a decrease in A_lim_ and the fluidity of monolayers were caused by an elevated temperature up to 27 °C (Figure 3A), and once again, the effect was stronger in the mutants than in the reference WT cultivar and concerned not only MGDG but the DGDG fraction as well. In both cases, 5 °C and 27 °C, the A_lim_ values corresponded well with a higher molar percentage of 18:3 in the MGDG fraction as well as a higher ratio of 18:3/18:2 and U/S. This correlation is consistent with expectations because linolenic acid, which contains three double bonds in the *cis* configuration, has the greatest impact on increasing the distance between the lipid hydrocarbon chains [30]. It is worth noting that in the MGDG fraction, present mainly in thylakoids, the higher values of membrane fluidity in the mutants at 5 °C and lower at 27 °C, compared to the WT Bowman, could be one of the reasons for the higher efficiency of photosystem II that was observed in the mutants [16]. According to Escribá et al. [31], even small changes in the lipid compositions can affect the physicochemical properties of the membrane, such as its fluidity and, as a result, affect the biochemical function of the signaling and transport proteins that are located in the membrane.

As mentioned, BR seems to be one of the hormones that regulate the biosynthesis of the main fatty acid—18:3 and/or its incorporation into the membranes. Such regulation may influence various physiological processes that are related to the membranes (such as the light reactions of photosynthesis), however, the question arises as to what significance this has for the frost tolerance or high-temperature tolerance of the whole plant that is acquired as a result of acclimation. Our work [16], showed that despite the metabolic disorders that differentiated the mutants from the WT Bowman, the mutants (after acclimation at 27 °C) had a higher tolerance to temperatures around 40 °C than the WT. In contrast, their frost tolerance (measured after acclimation at 5 °C) was lower than in WT Bowman. In our other studies [17,18], we were trying to explain this phenomenon by analyzing the changes in important membrane proteins. In the current study, we attempted to explain it by analyzing the physicochemical properties of the lipid membranes from plants that had been acclimated at 5 °C (thus hardened to frost) or acclimated at 27 °C (thus more tolerant to a much higher temperature).

The observed changes in membrane saturation, which were characterized by the ratio of the most common fatty acids that were present in the plant membranes, i.e., 18:3/18:2, were usually accompanied by changes in the physicochemical parameters in the model membrane system that had been obtained from the lipid fractions. The clearest correlation was observed in the monolayers of the lipids from plants acclimated at 5 °C. In the analyzed mutants, the parameters that were calculated for the monolayers showed that a higher unsaturation (18:3/18:2 and U/S, MGDG fraction, Figure 1A,B) was associated with a higher value of A_lim_—surface area per single lipid molecule (Figure 3A)—thereby illustrating a higher degree of membrane fluidity. Lower unsaturation (18:3/18:2 and U/S, PL fraction, Figure 1E,F) was associated with a decrease in the surface area per single lipid molecule (A_lim_, Figure 3G). While the lack of this regularity was observed in some cases, it can be explained by the fact that A_lim_ is also affected by other factors, such as the charges that are localized on the polar part of lipids. Moreover, as was mentioned above, an increase in the surface area per single lipid molecule (A_lim_) usually means a higher degree of the fluidity of the monolayer while a decrease of A_lim_ is connected to a lower degree of fluidity. It is believed that higher membrane fluidity is more beneficial for better frost tolerance [32,33]. As the analyzed mutants had a lower frost tolerance than the WT Bowman after acclimation at 5 °C [16], the obtained results may at least partly explain the reason for this. Only the monolayers of MGDG had higher A_lim_ values, and consequently a higher degree of fluidity, whereas the DGDG or PL monolayers which mainly build the plasma membrane did not. Based on this model study, it can be suspected that the natural cell membranes of the mutants also have a lower degree of fluidity than the WT Bowman membranes at 5 °C and that this could be one of the factors that influence the higher frost susceptibility of the mutants in comparison with the WT Bowman [16]. Interestingly, more membrane injuries (measured as electrolyte leakage) were reported by Qu et al. [34] and Eremina et al. [35] in the *Arabidopsis* BR-signaling mutants that had been exposed to temperatures of 0 °C and below, which also confirms the connection of BR to the membrane “adaptation” to this stress. 

Moreover, the mutants acclimated at 27 °C were less susceptible to heat stress (about 40 °C) than the WT [16]. In both mutants, A_lim_ for the MGDG and DGDG monolayers reached a lower value, than in the WT Bowman, which indicates a lower degree of fluidity. This feature is more desirable as an “adaptation” to high-temperature stress. It is worth mentioning that the mutants had lower membrane injuries after high-temperature exposure (estimated based on electrolyte leakage) than the WT [16]. To conclude, the changes leading to a lower degree of fluidity that were observed in the membranes of the analyzed mutants could be part of mechanisms that are associated with the improved tolerance of these mutants to the heat stress.

An analysis of the relationships between the other physicochemical parameters revealed that the pressure at which the monolayer collapses (π_coll_) and the compression modulus (Cs^−1^) in mutants were also changed when compared to the WT Bowman and were dependent on the temperature of plant growth/acclimation (Figure 3B,C,E,F,H,I and Figure S1A–I). The values of these parameters provide additional information on the stability and flexibility of the monolayers as a result of the strength of the interactions that occur between the saturated and unsaturated FAs [29,30]. The increase in the π_coll_ value (the value of the surface pressure at which a layer collapses) may result from better geometric alignment of the lipid particles (usually for saturated acids), but it may also be modified by the electrostatic interactions between polar lipid parts. The value of this parameter was most often lower after acclimation at 5 °C and 27 °C for the mutant with BR-signaling disorders (for all of the fractions) and in the galactolipid fraction MGDG for the BR-biosynthesis mutant.

All of the changes that occur in the physicochemical and structural state of membranes (as a result of modification in the lipid composition for membrane acclimation to lower/higher temperatures) can also influence the possibility of the interactions and locations of various compounds (sterols, steroid hormones, etc.) in the membranes. The fact that the mutants, compared to the WT, had altered physicochemical and structural parameters characterizing the membranes shows how wide and multidirectional the impact of brassinosteroids can be on the membrane-dependent physiological processes.

Sometimes the directions of changes in the parameters studied were different in the mutant with BR deficit in comparison to a mutant with the BR-signaling disorder. A possible explanation can be that the BR-deficient mutant, however, produces low amounts of BR which still can interact with the BR receptor to induce a physiological responses to BR (i.e. connected to lipid biosynthesis). In the case of the mutant with BR-signaling disorder, despite BR overproduction (resulting from the feedback mechanism), signal perception is disturbed and the physiological response is also disturbed. 

## 5. Conclusions 

Brassinosteroids seem to be one of the players that regulate fatty acid biosynthesis or their transport/incorporation into the cell membranes. The BR-biosynthesis and BR-signaling mutants of barley were characterized by a temperature-dependent altered molar percentage of fatty acids (from 14:0 to 20:1) in their galactolipid and phospholipid fractions, which suggests that BR play a role in lipid management, although the mechanism of this regulation requires further studies. The fact that the BR-biosynthesis and BR-signaling mutants had altered physicochemical parameters of their membranes, compared to the WT, shows that BR may have a multidirectional impact on the membrane-dependent physiological processes.

## Figures and Tables

**Table 1:** Composition of the fatty acids from the three classes of lipids (MGDG, DGDG, PL) that were isolated from the barley WT Bowman and its BW084 and BW312 mutants cultured at 20 °C and then acclimated at 5 °C or 27 °C. Any significant differences between WT Bowman and its mutants (Duncan test, *p* ≤ 0.05) for each temperature, lipid fraction, and fatty acid (separately) are indicated by different letters.

Temperature	Plants	Fatty Acids [mol %]										
		14:0	16:0	16:1n-9	16:1n-7	18:0	18:1n-9	18:1n-7	18:2n-6	18:3n-3	20:0	20:1
**MGDG**												
20 °C	Bowman	0.321 ^b^	5.270 ^c^	0.049 ^b^	0.069 ^a^	2.695 ^c^	1.208 ^b^	0.190 ^b^	4.458 ^b^	85.335 ^a^	0.075 ^c^	0.046 ^a^
20 °C	BW084	0.584 ^a^	8.271 ^a^	0.094 ^a^	0.071 ^a^	4.201 ^a^	1.340 ^a^	0.252 ^a^	5.216 ^a^	79.399 ^c^	0.121 ^a^	0.049 ^a^
20 °C	BW312	0.298 ^b^	6.011 ^b^	0.035 ^c^	0.061 ^a^	3.040 ^b^	1.005 ^c^	0.207 ^b^	5.180 ^a^	83.800 ^b^	0.088 ^b^	0.031 ^b^
5 °C	Bowman	0.257 ^b^	7.380 ^a^	0.041 ^b^	0.046 ^a^	3.900 ^a^	1.157 ^a^	0.191 ^a^	3.483 ^a^	82.928 ^c^	0.152 ^a^	0.078 ^a^
5 °C	BW084	0.233 ^b^	5.128 ^c^	0.032 ^b^	0.052 ^a^	3.767 ^b^	0.770 ^c^	0.173 ^a^	2.648 ^c^	86.709 ^a^	0.141 ^a^	0.034 ^c^
5 °C	BW312	0.293 ^a^	5.702 ^b^	0.057 ^a^	0.056 ^a^	3.288 ^c^	1.066 ^b^	0.195 ^a^	3.043 ^b^	85.840 ^b^	0.093 ^b^	0.056 ^b^
27 °C	Bowman	0.387 ^c^	7.858 ^b^	0.031 ^c^	0.067 ^a^	4.874 ^c^	1.017 ^b^	0.211 ^a^	6.640 ^b^	78.427 ^a^	0.147 ^b^	0.030 ^a^
27 °C	BW084	0.484 ^b^	9.282 ^a^	0.074 ^b^	0.069 ^a^	6.646 ^a^	1.046 ^b^	0.222 ^a^	6.600 ^b^	74.951 ^b^	0.196 ^a^	0.026 ^a^
27 °C	BW312	0.582 ^a^	9.369 ^a^	0.222 ^a^	0.054 ^b^	5.767 ^b^	1.172 ^a^	0.205 ^a^	7.425 ^a^	74.527 ^c^	0.159 ^b^	0.030 ^a^
**DGDG**												
20 °C	Bowman	0.228 ^c^	13.474 ^a^	0.103 ^b^	0.085 ^ab^	1.998 ^a^	1.606 ^b^	0.354 ^ab^	3.821 ^c^	77.904 ^a^	0.028 ^b^	0.061 ^a^
20 °C	BW084	0.326 ^a^	13.532 ^a^	0.106 ^b^	0.091 ^a^	1.899 ^b^	1.733 ^a^	0.370 ^a^	4.228 ^b^	77.151 ^b^	0.040 ^a^	0.061 ^a^
20 °C	BW312	0.246 ^b^	12.920 ^b^	0.189 ^a^	0.072 ^b^	1.689 ^c^	1.524 ^c^	0.336 ^b^	4.783 ^a^	77.803 ^a^	0.030 ^b^	0.057 ^a^
5 °C	Bowman	0.178 ^a^	12.986 ^a^	0.124 ^a^	0.084 ^a^	1.582 ^b^	1.364 ^a^	0.341 ^a^	2.678 ^c^	80.131 ^c^	0.051 ^b^	0.075 ^a^
5 °C	BW084	0.140 ^b^	11.694 ^b^	0.037 ^c^	0.075 ^b^	1.167 ^c^	1.022 ^c^	0.314 ^a^	3.070 ^a^	82.039 ^a^	0.046 ^b^	0.070 ^a^
5 °C	BW312	0.132 ^b^	11.728 ^b^	0.054 ^b^	0.064 ^c^	1.809 ^a^	1.171 ^b^	0.320 ^a^	2.852 ^b^	81.413 ^b^	0.083 ^a^	0.047 ^b^
27 °C	Bowman	0.320 ^b^	17.561 ^b^	0.058 ^c^	0.082 ^a^	2.630 ^a^	1.446 ^a^	0.344 ^a^	6.093 ^b^	70.943 ^b^	0.038 ^a^	0.056 ^a^
27 °C	BW084	0.298 ^b^	15.433 ^c^	0.065 ^b^	0.085 ^a^	2.217 ^b^	1.077 ^b^	0.328 ^a^	5.988 ^b^	73.967 ^a^	0.041 ^a^	0.042 ^b^
27 °C	BW312	0.562 ^a^	18.933 ^a^	0.220 ^a^	0.071 ^b^	2.595 ^a^	1.457 ^a^	0.331 ^a^	6.987 ^a^	68.074 ^c^	0.057 ^a^	0.026 ^c^
**PL**												
20 °C	Bowman	0.165 ^b^	22.483 ^b^	0.045 ^b^	0.070 ^a^	1.200 ^b^	3.839 ^a^	0.427 ^b^	20.456 ^b^	50.346 ^a^	0.117 ^b^	0.220 ^a^
20 °C	BW084	0.169 ^b^	22.393 ^b^	0.054 ^a^	0.070 ^a^	1.264 ^ab^	3.654 ^b^	0.506 ^a^	22.228 ^a^	48.504 ^c^	0.174 ^a^	0.221 ^a^
20 °C	BW312	0.198 ^a^	23.345 ^a^	0.040 ^c^	0.069 ^a^	1.305 ^a^	2.661 ^c^	0.404 ^b^	22.005 ^a^	49.017 ^b^	0.124 ^b^	0.168 ^b^
5 °C	Bowman	0.136 ^a^	22.258 ^b^	0.028 ^a^	0.052 ^b^	1.064 ^a^	2.409 ^a^	0.329 ^a^	16.411 ^c^	56.357 ^a^	0.087 ^c^	0.231 ^a^
5 °C	BW084	0.119 ^b^	23.162 ^a^	0.034 ^a^	0.061 ^a^	1.071 ^a^	1.992 ^b^	0.329 ^a^	21.462 ^a^	50.779 ^c^	0.151 ^a^	0.160 ^c^
5 °C	BW312	0.108 ^b^	22.730 ^a^	0.019 ^b^	0.048 ^b^	1.113 ^a^	2.393 ^a^	0.348 ^a^	18.826 ^b^	53.552 ^b^	0.112 ^b^	0.196 ^b^
27 °C	Bowman	0.246 ^a^	25.844 ^a^	0.070 ^a^	0.072 ^b^	1.640 ^a^	3.585 ^a^	0.481 ^a^	20.618 ^b^	46.404 ^c^	0.106 ^c^	0.139 ^a^
27 °C	BW084	0.246 ^a^	24.314 ^b^	0.073 ^a^	0.083 ^a^	1.409 ^c^	2.493 ^c^	0.435 ^b^	19.856 ^c^	49.991 ^a^	0.143 ^b^	0.096 ^b^
27 °C	BW312	0.227 ^b^	24.122 ^b^	0.072 ^a^	0.069 ^b^	1.523 ^b^	2.691 ^b^	0.397 ^c^	22.808 ^a^	46.957 ^b^	0.156 ^a^	0.101 ^b^

## Supplementary Materials

The following can be found at https://www.mdpi.com/2073-4409/9/5/1125/s1. **Figure S1**. Changes in the values of the A_lim_, π_coll_ and C_s_^−1^ parameters for the barley brassinosteroid mutants (BW084 and BW312) and the WT Bowman growing at 5 °C and 27 °C in relation to 20 °C (when 20 °C is expressed as 100%). (**A**, **B**, **C**) Data calculated for monolayers of monogalactosyldiacylglycerols (MGDG); (**D**, **E**, **F**) Data calculated for monolayers of digalactosyldiacylglycerols (DGDG); (**G**, **H**, **I**) Data calculated for monolayers of phospholipids (PL).
