# Supplementary material for: The Impact of Mutations in the HvCPD and HvBRI1 Genes on the Physicochemical Properties of the Membranes from Barley Acclimated to Low/High Temperatures
Source: Cells. 2020 May 1;9(5):1125. doi: 10.3390/cells9051125 (PMC7290739; doi:10.3390/cells9051125)

Figure S1. Changes in the values of the A_{lim}, π_{coll} and C_s^{-1} parameters for the barley brassinosteroid mutants (BW084 and BW312) and the WT Bowman growing at 5°C and 27°C in relation to 20 °C (when 20°C is expressed as 100%). (**A**, **B**, **C**) Data calculated for monolayers of monogalactosyldiacylglycerols (MGDG); (**D**, **E**, **F**) Data calculated for monolayers of digalactosyldiacylglycerols (DGDG); (**G**, **H**, **I**) Data calculated for monolayers of phospholipids (PL).

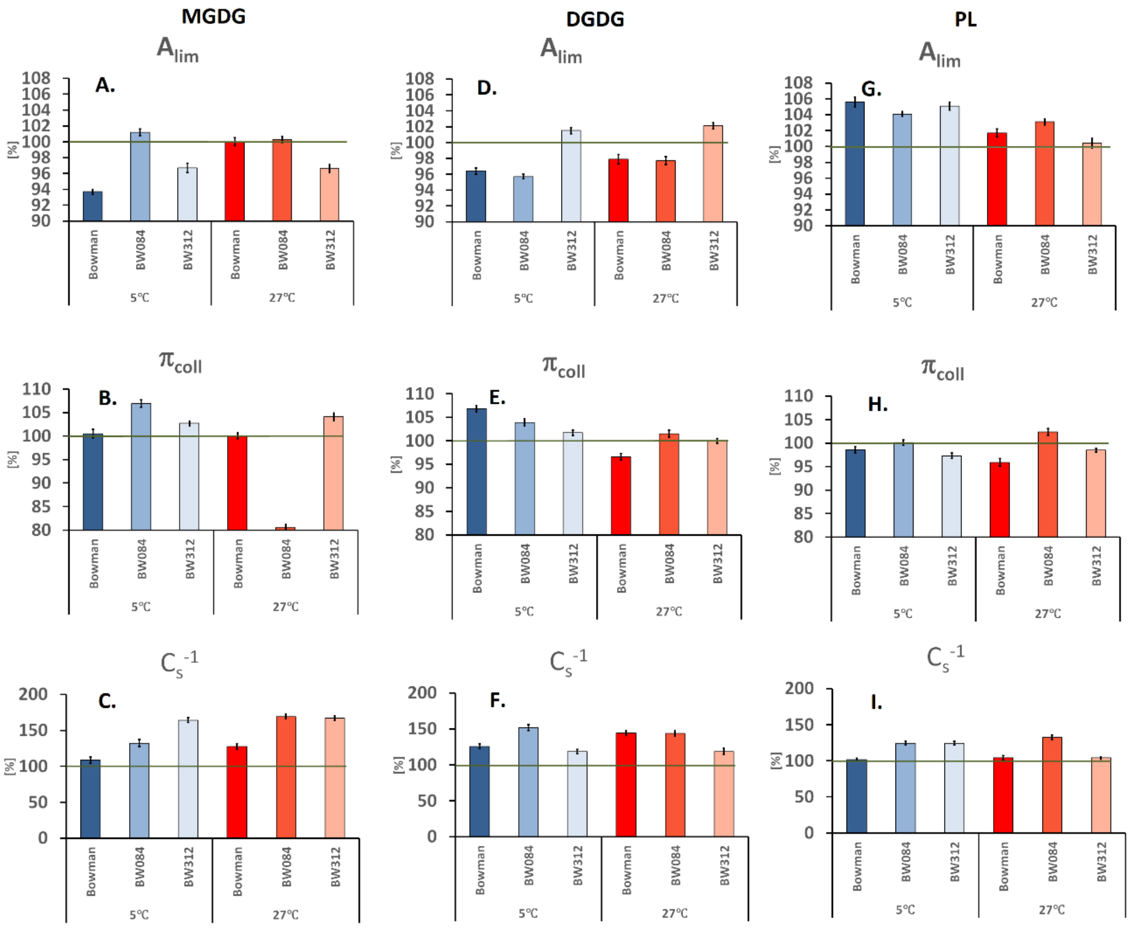

Supplement: Supplementary file 1 [file cells-09-01125-s001.pdf]
